# DNM3OS Enhances the Apoptosis and Senescence of Spermatogonia Associated with Nonobstructive Azoospermia by Providing miR-214-5p and Decreasing E2F2 Expression

**DOI:** 10.1155/2023/1477658

**Published:** 2023-12-20

**Authors:** Rui Hua, Qingjun Chu, Feiyan Guo, Qinjie Chen, Maocai Li, Xuan Zhou, Yongtong Zhu

**Affiliations:** Center for Reproductive Medicine, Department of Obstetrics and Gynecology, Nanfang Hospital, Southern Medical University, Guangzhou, China

## Abstract

**Background:**

Nonobstructive azoospermia (NOA) is a complex disease characterized by the spermatogenic dysfunction of testicular tissues. The roles played by long noncoding RNAs (lncRNAs) in NOA pathogenesis have not been extensively studied.

**Methods:**

Microarray assays were performed on samples of testicular biopsy tissue obtained from patients with NOA for the purpose of identifying differentially expressed lncRNAs and messenger RNA (mRNA) transcripts, and the results were verified by quantitative real-time polymerase chain reaction. Mouse-derived GC-1 spermatogonia (spg) cells undergoing treatment with Adriamycin (ADR) were used to investigate the biological functions of the selected lncRNAs *in vitro*. The target microRNAs (miRNAs) of lncRNAs and the target mRNAs of miRNAs were predicted by a bioinformatics analysis. Functional studies performed using the CCK-8 assay, EdU incorporation assay, apoptosis detection, and senescence-associated *β*-galactosidase (SA-*β*-Gal) staining were conducted using GC-1 spg cells.

**Results:**

Totals of 2,652 lncRNAs and 2,625 mRNAs were found to be differentially expressed in the testicular tissue of NOA patients when compared with patients in a control group. Dynamin 3 opposite strand (DNM3OS) was a provider of pe-miR-214-5p that positively regulates miR-214-5p expression in GC-1 spg cells. The E2 factor (E2F) family of transcription factor 2 (E2F2) was initially predicted and subsequently verified to be a downstream gene of miR-214-5p. E2F2 expression was upregulated after DNM3OS knockdown in ADR-treated GC-1 spg cells. Moreover, knockdown of either DNM3OS or miR-214-5p significantly alleviated ADR-induced decreases in cellular activity and proliferation, as well as increases in apoptosis and senescence of mouse spermatogonial GC-1 spg cells.

**Conclusions:**

DNM3OS was found to regulate the apoptosis and senescence of spermatogonia by providing miR-214-5p and decreasing E2F2 expression, suggesting it as a novel target for gene therapy of male infertility.

## 1. Introduction

Azoospermia is described as the absence of sperm in ejaculation and is classified as either obstructive azoospermia (OA) or nonobstructive azoospermia (NOA). Azoospermia affects 8% of couples worldwide and 10%–15% of infertile men [1, 2]. In contrast to OA with intact spermatogenesis [3], NOA is characterized by the severe spermatogenic dysfunction of testicular tissues caused by extreme genetic heterogeneity [4, 5]. The current first-line treatment for NOA consists of spermatozoa retrieval after microdissection testicular sperm extraction, and the second choice is *in vitro* fertilization using intracytoplasmic sperm injection [6, 7]. However, failed sperm retrieval, mainly ascribed to a lack of accurate indicators, often produces emotional and financial burdens for infertile couples [8]. Considering that NOA is a highly heterogeneous condition with a broad genetic basis, it is important to explore the molecular pathogenesis of NOA.

Noncoding RNAs (ncRNAs) are classified into categories of long ncRNAs (lncRNAs) with >200 nucleotides (nts) and sncRNAs with <200 nts [9]. LncRNAs have received increased attention for the regulatory role they play in male infertility by participating in the self-renewal, proliferation, and differentiation of spermatogonial stem cells [10, 11]. A previous study reported that the levels of lncRNA-Gm2044 were elevated in spermatocytes and suppressed the proliferation of mouse spermatogonia [12]. Hu et al. [13] constructed a transgenic mouse model and used it to show that lncRNA-Gm2044 levels were elevated in NOA and that an elevated level of lncRNA-Gm2044 partially impaired spermatogenesis [13]. Furthermore, lncRNA-linc00467 was found to serve as a ceRNA that influences male gamete generation by altering the levels of *Lrguk* and *Tdrd6* expression in NOA [14]. LncRNA033862 [15] and lncRNA AK015322 [16] have been suggested to be crucial for maintaining the proliferation and survival of spermatogonia stem cells (SSCs).

Among the sncRNAs, microRNAs (miRNAs, 18–25 nts in length) bind to specific sequences in the 3′-untranslated region (UTR) of their downstream genes and cause its protein expression to be blocked [17]. As upstream regulators of miRNAs, lncRNAs modulate the levels or functions of miRNAs by serving as competing endogenous RNAs that share miRNA-binding sites and thereby alter the expression of specific targeted messenger RNAs (mRNAs) [18, 19]. Abnormal expression of various miRNAs has been reported to occur in the reproductive system and plays an important role in the apoptosis and self-renewal of SSCs. A previous study reported that the levels of hsa-miR-30a-5p were higher in men with NOA than in control individuals [20]. Functionally, has-miR-449a suppresses the proliferation of mouse spermatogonia by inhibiting CEP55 expression [21]. Moreover, miRNA-122-5p inhibits calcineurin binding protein-like (CBL) expression and promotes the proliferation of human SSCs [22]. Our understanding of how the interactions of lncRNAs, miRNAs, and mRNAs are associated with NOA pathogenesis has remained limited until now.

The E2 factor (E2F) family is composed of 8 members (E2F1–8) [23, 24], and they are able to regulate a variety of cellular processes [25, 26]. E2F2 regulates G1/S transition and cell cycle progression through S phase to promote cell transformation [27]. In a variety of diseases, it also enhances the proliferation of nonproliferative tumor cells by influencing the cell cycle [28, 29].

In the present study, microarray analyses were performed to examine the expression profiles of lncRNAs and mRNAs in patients with NOA. After screening and validation of several top highly altered markers, we further performed *in vitro* experiments to investigate the roles of certain lncRNA/miRNA/mRNA axes in regulating the apoptosis and senescence of spermatogonia associated with NOA.

## 2. Materials and Methods

### 2.1. Collection of Tissue Samples

Samples of testicular biopsy tissue were obtained from patients with NOA (*n* = 9) and nine urology patients undergoing orchiectomy as controls (*n* = 9) at the Nanfang Hospital of Southern Medical University. All men with NOA were diagnosed after a complete history and physical examination, including an ultrasound of the scrotum (the clinical information is summarized in Table 1). The diagnosis of azoospermia is based on the fifth edition of the World Health Organization laboratory manual “Examination and Treatment of Human Semen” [30]. The testicular histology of NOA patients was characterized as hypospermatogenesis and germ cell mature arrest. NOA patients with a varicocele, Y chromosome microdeletion, or chromosomal abnormality were excluded from the study. The diagnosis of NOA is made after a complete evaluation by an experienced andrologist using all of the above information. Considering that it is impractical to obtain testicular samples from volunteers with known normal fertility, we selected urology patients without meiotic defects or infertility and who had not received adjuvant hormonal therapy prior to orchiectomy. The study protocol was approved by the Institutional Review Board of Nanfang Hospital, Southern Medical University, and all patients provided their written informed consent for study participation (no. NFEC-2019-219).

### 2.2. LncRNA and mRNA Expression Microarray

TRIzol reagent (Invitrogen, Carlsbad, CA, USA) was used to isolate the total RNA, and its quality was determined using a NanoDrop 2000 spectrophotometer (NanoDrop Technologies, Wilmington, DE, USA). Subsequently, an aliquot of total RNA (100 ng) derived from testicular samples was labeled using a Quick Amp Labeling Kit (Agilent Technologies, Santa Clara, CA, USA). Next, each sample was hybridized with an Agilent Gene Expression Hybridization Kit on a Microarray Hybridization Chamber. After washing, the hybrid signal value was scanned with an Agilent G2565BA microarray scanner (Agilent Technologies). The raw data were extracted and then normalized according to quartiles and processed using the limma R package. The genes were analyzed using highly reliable public transcriptome databases (GENCODE, Noncode, LNCipedia, Ensembl, Lncrnadb, and UCSC). LncRNAs and mRNAs with differential expression (NOA vs. control) were identified using Student's *t*-test with a significance cutoff value of *p* <  0.05 and an absolute fold-change value > 2.0. The top 20 distinguishable upregulated and downregulated lncRNAs were further displayed by hierarchical clustering. Differences in mRNA expression patterns between samples were illustrated using the heatmap package.

### 2.3. Bioinformatics Analysis

Differentially expressed mRNAs were input into the DAVID database (https://david.ncifcrf.gov) for the purpose of performing a gene ontology (GO) functional and Kyoto Encyclopedia of Genes and Genomes (KEGG) pathway enrichment analysis with a cutoff *p*-value < 0.05. The GO-related terms consisted of biological process, cellular component, and molecular function (MF).

### 2.4. Cell Treatment

Human male germ TCAM-2 cells and mouse-derived GC-1 spermatogonia (spg) cells were cultured in RPMI-1640 medium (Life Technologies, Paisley, UK) supplemented with 10% fetal bovine serum (FBS) and 1% penicillin/streptomycin at 37°C in a 5% CO_2_ atmosphere. TCAM-2 and GC-1 spg cells were stimulated with either lipopolysaccharide (LPS) (10 *μ*g/mL, L2630, Sigma-Aldrich, St. Louis, MO, USA) or Adriamycin (ADR, 0.5 *μ*M, D1515, Sigma-Aldrich) for 24 hr.

### 2.5. Cell Transfection

An miR-214-5p inhibitor, a negative control (NC), miR-214-5p mimics, and NC mimics, as well as small interfering RNA-targeting DNM3OS (si-DNM3OS) and a si-NC, were synthesized by RiboBio (Guangzhou, China). GC-1 spg cells were cocultured with si-DNM3OS and si-NC and then cultured in an incubator for 24 hr with 0.5 *μ*M ADR to investigate the effect of DNM3OS knockdown. To perform miR-214-5p knockdown, GC-1 spg cells were cocultured with the inhibitor or NC for 24 hr prior to treatment with 0.5 *μ*M ADR. All transfections were performed using Lipofectamine 2,000 reagent (Invitrogen).

### 2.6. Cell Activity Detection

The activity of GC-1 spg cells from different groups was assessed using the CCK-8 assay. In brief, GC-1 spg cells from different groups were plated in 96-well plates. After incubation, CCK-8 solution (10 *μ*L, Dojindo, Kumamoto, Japan) was added to each well, and the cells were cultured for 2 hr at 37°C. The absorbance (450 nm) of each sample well was measured with a microplate reader (Thermo Fisher Scientific, Waltham, MA, USA).

### 2.7. Cell Proliferation Assay

GC-1 spg cells were added to 12-well plates (4 × 10^5^ cells per well) and then cultured with 100 *μ*M EdU (RiboBio). Next, the cells were fixed with paraformaldehyde (4%) for 30 min and permeabilized with Triton-100 (0.25%) for 10 min. The cells were then rinsed three times with phosphate-buffered saline (PBS) and stained with DAPI (4′,6-diamidino-2-phenylindole, 5 *μ*g/mL) in the dark. Subsequently, cell fluorescence was evaluated under a fluorescence microscope, and images were acquired at ×100 magnification.

### 2.8. Cell Apoptosis Detection

Approximately 5 × 10^5^ GC-1 spg cells were harvested via trypsinization and suspended in the buffer. Next, the cells were stained with FITC-Annexin V and PI solution (5 *μ*L, BD Biosciences, San Jose, CA, USA) for 5 min in the dark at room temperature. Finally, a FACS Calibur flow cytometer (BD Biosciences) equipped with WinMDI V2.9 software was used to analyze the stained cells for the presence of apoptotic cells.

### 2.9. Cell Senescence Assay

The senescence of GC-1 spg cells was visualized by staining with a Senescence-Associated *β*-Galactosidase (SA-*β*-Gal) kit (Beyotime Institute of Biotechnology, Shanghai, China). In brief, processed GC-1 spg cells were treated with PBS fixation solution containing 2% formaldehyde/0.2% glutaraldehyde for 5 min at room temperature and then washed three times with PBS. After an overnight incubation in the SA-*β*-Gal staining reagent at 37°C, cells with positive SA-*β*-Gal staining were detected under a microscope. The images were then amplified 100-fold using an electron microscope.

### 2.10. Luciferase Reporter Assay

Luciferase reporter assays were performed to determine whether E2F2 contains a binding site for miR-214-5p. After amplifying the fragment of E2F2 containing the suspected miR-214-5p-binding site, psiCHECK-2 vectors (Promega, Madison, WI, USA) were constructed for the purpose of generating wild-type (WT) and mutant (MUT) E2F2. GC-1 spg cells were transfected with a plasmid vector carrying either WT-E2F2 or MUT-E2F2 and miR-214-5p mimics or NC mimics. After 48 hr of culture, the levels of dual-luciferase activity were measured, and the data obtained for firefly activity were normalized to Renilla activity.

### 2.11. Quantitative Real-Time Polymerase Chain Reaction (qPCR)

TRIzol reagent (Invitrogen) was used to extract total RNA, and TransScript First-Strand cDNA Synthesis SuperMix (TransGen Biotech, Beijing, China) was used for reverse transcription. DNA amplification was performed by using SYBR Green Taq Mix (Takara, Tokyo, Japan) on an ABI 7500 Fast Real-Time PCR System (ABI, Foster City, CA, USA). The conditions used for PCR were as follows: 95°C for 2 min, 95°C for 15 s (40 cycles), and 60°C for 30 s. The primer sequences are shown in Table 2. Relative levels of *DNM3OS*, *miRNAs*, and *E2F2* gene expression were calculated using the 2^−*ΔΔ*Ct^ method. U6 and GAPDH served as internal references for miRNAs and mRNAs, respectively.

### 2.12. Western Blotting

The total protein in each sample was extracted using ice-cold RIPA lysis buffer (Thermo Fisher Scientific), and the protein concentration in each extract was quantified using the BCA assay (Beyotime Institute of Biotechnology). Next, a 10 *µ*g sample of total protein from each group was separated by sodium dodecyl sulfate-polyacrylamide gel electrophoresis (SDS–PAGE), and the protein bands were electrophoretically transferred onto polyvinylidene fluoride (PVDF) membranes (Millipore, Burlington, MA, USA), which were subsequently blocked with 5% BSA in tris-buffered saline with tween (TBST) for 4 hr at 4°C. The membranes were then incubated with anti-E2F2 and anti-*β*-actin antibodies (Abcam, Cambridge, MA, USA) for 2 hr; after which, they were washed three times with TBST and incubated with a horseradish peroxidase (HRP)-conjugated secondary antibody at room temperature for 2 hr. Finally, the protein staining signals were detected by enhanced chemiluminescence (Amersham, Bucks, UK).

### 2.13. Statistical Analysis

All quantitative data were analyzed using GraphPad Prism 8.0 software (GraphPad Software, La Jolla, CA). All results were calculated using data obtained from three independent experiments and are expressed as a mean value ± standard deviation. Student's *t*-test was used to analyze the differences between the two groups. One-way analysis of variance followed by Dunnett's post hoc test or Tukey's post hoc test was used to make differential comparisons among multiple groups. A *p*-value < 0.05 was considered to be statistically significant.

## 3. Results

### 3.1. Microarray and Bioinformatics Analysis of NOA Tissues

**Figure 1:**
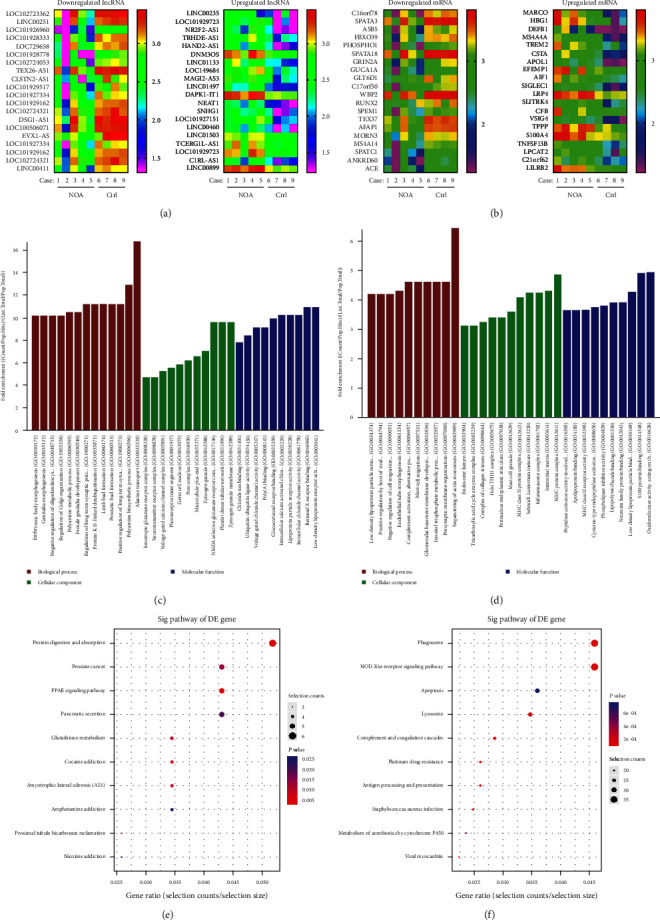
Microarray data and bioinformatics analysis of NOA tissues. Heatmap of the top 20 downregulated and upregulated lncRNAs (a), as well as the top 20 downregulated and upregulated mRNAs in NOA tissues when compared with normal tissues (b). The downregulated (c) and upregulated (d) mRNAs from GO enrichment analyses that are related to biological processes (BP), cellular components (CC), and molecular functions (MF). KEGG pathway analysis of downregulated (e) and upregulated (f) mRNAs. GO, gene ontology, KEGG, Kyoto Encyclopedia of Genes and Genomes.

Our microarray analyses revealed the lncRNA and mRNA expression profiles of human NOA and normal tissues. Totals of 2,652 lncRNAs (1,656 increased/996 decreased genes) and 2,625 mRNAs (2,192 increased/433 decreased genes) were found to be differentially expressed in NOA tissues when compared with normal tissues. Heatmaps were created to display the top 20 changed lncRNAs (Figure 1(a)), as well as the top 20 changed mRNAs (Figure 1(b)). Next, GO analyses showed that the downregulated mRNAs might be associated with polyamine biosynthetic process (GO: 0006596), alanine transport (GO: 0032328), N-methyl-D-aspartate (NMDA) selective glutamate receptor complex (GO: 0017146), platelet dense tubular network (GO: 0031094), zymogen granule membrane (GO: 0042589), and low-density lipoprotein receptor activity (GO: 0005041) (Figure 1(c)), while the upregulated mRNAs were involved in sequestering of actin monomers (GO: 0042989), major histocompatibility complex (MHC) protein complex (GO: 0042611), S100 protein binding (GO: 0044548) and oxidoreductase activity, acting on the CH─CH group of donors, and nicotinamide adenine dinucleotide (NAD) or nicotinamide adenine dinucleotide phosphate (NADP) as an acceptor (GO: 0016628) (Figure 1(d)). Subsequent KEGG pathway analyses indicated that the downregulated mRNAs participated in protein digestion and absorption, proximal tubule bicarbonate reclamation, peroxisome proliferator-activated receptor (PPAR) signaling pathway, cocaine addiction, and amyotrophic lateral sclerosis (Figure 1(e)), while the upregulated mRNAs were involved in phagosome, nucleotide-binding oligomerization domain (NOD)-like receptor signaling pathway, apoptosis, and lysosome, complement, and coagulation cascades (Figure 1(f)).

### 3.2. Validation of LncRNAs with Differential Expression

**Figure 2:**
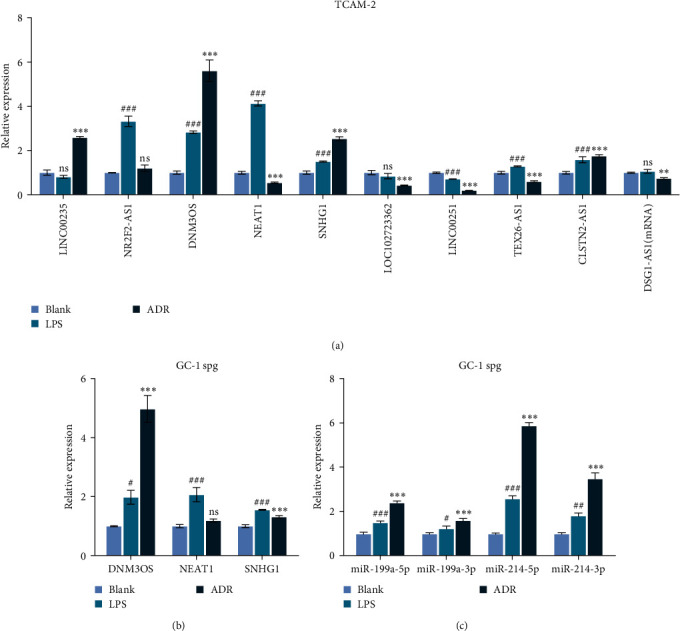
Validation of lncRNAs from microarray data. (a) Five upregulated and downregulated lncRNAs selected from the 20 top differentially expressed lncRNAs were analyzed for their expression levels in TCAM-2 cells undergoing treatment with LPS or ADR alone. (b) The levels of DNM3OS, NEAT1, and SNHG1 expression in GC-1 spg cells treated with LPS or ADR alone were determined by quantitative real-time PCR analysis. (c) The expression levels of DNM3OS precursor transcripts, including miR-199a-5p, miR-199a-3p, miR-214-5p, and miR-214-3p, were measured in GC-1 spg cells after treatment with LPS or ADR alone. Data represent the mean value ± SD. ^#^*p* < 0.05, ^##^*p* < 0.01, ^###^*p* < 0.001 represents LPS vs. blank;  ^*∗∗*^*p* < 0.01,  ^*∗∗∗*^*p* < 0.001 represents ADR vs. blank.

To confirm the microarray results, five upregulated and five downregulated lncRNAs from the 20 top changed lncRNAs were selected, and their transcription levels were detected in TCAM-2 cells. Consistent with the microarray analysis, the expression levels of LINC00235, NR2F2-AS1, DNM3OS, NEAT1, and SNHG1 were significantly upregulated, while the expression levels of LOC102723362, LINC00251, TEX26-AS1, CLSTN2-AS1, and DSG1-AS1 were significantly downregulated in TCAM-2 cells undergoing treatment with LPS or ADR alone (Figure 2(a)). Subsequently, we analyzed three lncRNAs (DNM3OS, NEAT1, and SNHG1) that were highly expressed in GC-1 spg cells. qPCR data showed that the levels of both DNM3OS and NEAT1 expression were markedly elevated in GC-1 spg cells that had been stimulated with LPS or ADR alone (Figure 2(b)). DNM3OS expression showed the largest increase and was thus selected for subsequent analysis. Considering that *DNM3OS*, as a miRNA-encoding gene, serves as the precursor transcript of miR-199 a and miR-214 [31], we further analyzed the expression levels of miR-199a-5p/3p and miR-214-5p/3p. As shown in Figure 2(c), all four miRNAs were significantly upregulated in GC-1 spg cells that had been stimulated with either LPS or ADR alone, and miR-214-5p showed the greatest increase in expression.

### 3.3. Knockdown of DNM3OS Decreased the Apoptosis and Senescence of Spermatogonia Induced by ADR Treatment

**Figure 3:**
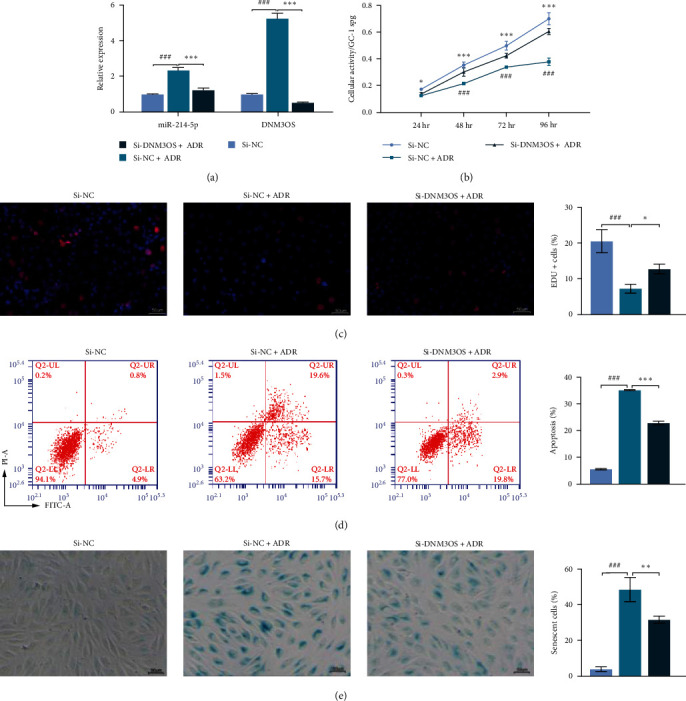
Knockdown of DNM3OS decreased the apoptosis and senescence of spermatogonia induced by ADR treatment. GC-1 spg cells were transfected with si-DNM3OS or si-NC, followed by 24 hr of incubation with 0.5 *μ*M ADR. (a) The levels of DNM3OS and miR-214-5p expression were determined by quantitative real-time PCR. (b) Cellular activity was determined by the CCK-8 assay. (c) EdU incorporation assays were performed to assess cell proliferation ability. The actual length of the scale in the images is 50 *μ*m. (d) Apoptotic cells were identified by flow cytometry with Annexin V-PI double staining. (e) Senescence-associated *β*-galactosidase (SA-*β*-gal) staining was used to evaluate cell senescence. The actual length of the scale in the images is 50 *μ*m. Data represent the mean value ± SD. ^###^*p* < 0.001 represents si-NC vs. si-NC + ADR;  ^*∗*^*p* < 0.05,  ^*∗∗∗*^*p* < 0.001 represents si-NC + ADR vs. si-DNM3OS + ADR.

Because it was difficult to stably culture human male germ TCAM-2 cells *in vitro*, we selected mouse-derived GC-1 spg cells to explore how DNM3OS regulates ADR-induced spermatogonia. First, qPCR results suggested that the ADR-induced upregulation of DNM3OS and miR-214-5p in ADR-stimulated GC-1 spg cells was significantly reversed after transfection with si-DNM3OS (Figure 3(a)). Next, results from CCK-8 assays showed that the impaired cellular activity of GC-1 spg cells caused by ADR treatment was obviously alleviated after DNM3OS knockdown (Figure 3(b)). EdU incorporation assays demonstrated that si-DNM3OS transfection obviously attenuated the decreased proliferation ability of GC-1 spg cells induced by ADR treatment (Figure 3(c)). Flow cytometry further confirmed that the ADR-induced increase in apoptotic GC-1 spg cells was dramatically suppressed after DNM3OS knockdown (Figure 3(d)). Moreover, silencing of DNM3OS decreased the ADR-induced senescence, as reflected by SA-*β*-Gal activity in GC-1 spg cells (Figure 3(e)).

### 3.4. DNM3OS Negatively Regulated E2F2 Expression by Providing Pre-MiR-214-5p

**Figure 4:**
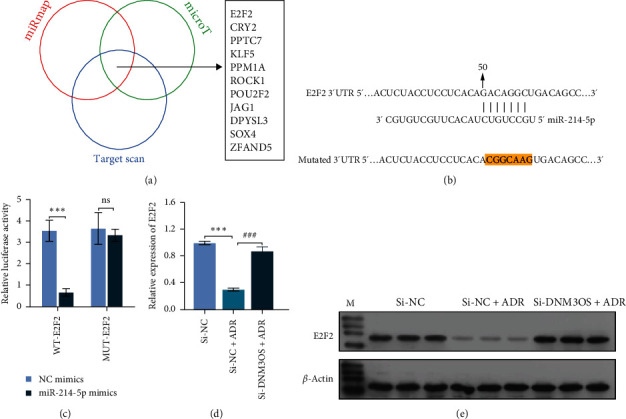
DNM3OS negatively regulated E2F2 expression by competitively binding to miR-214-5 p. (a) The TargetScan, microT, and miRmap databases were used to create a Venn diagram, and the intersection predicted 11 overlapping target genes of miR-214-5p. (b) Schematic representation of miR-214-5p target sites in the 3′-UTR of E2F2 mRNA and the 3′-UTR of the E2F2 mutant mRNA containing altered nucleotides in the putative target site (E2F2-3′-UTR-MUT). (c) MiR-214-5p mimics or NC mimics were simultaneously transfected into GC-1 spg cells containing the wild-type or mutant E2F2-3′-UTR, and luciferase reporter gene assays were performed to measure luciferase activity in the cells.  ^*∗∗∗*^*p* < 0.001 represents NC mimics *vs*. miR-214-5 p mimics; (d) and (e) E2F2 expression at the mRNA and protein levels was determined in GC-1 spg cells that had been transfected with si-DNM3OS, and then incubated for 24 hr with 0.5 *μ*M ADR.  ^*∗∗∗*^*p* < 0.001 represents si-NC vs. si-NC + ADR; ^###^*p* < 0.001 represents si-NC + ADR vs. si-DNM3OS + ADR; data represent the mean value ± SD.

To identify the downstream regulators of miR-214-5p, we utilized three publicly available algorithms (TargetScan, microT, and miRmap) to identify miR-214-5p targets in mice. The intersection of the downstream genes predicted by all three databases consisted of 11 overlapping target genes (Figure 4(a)). After integrating the microarray and published data (Table 3), E2F2 was selected as a potential downstream target gene of miR-214-5p with a specific binding site (Figure 4(b)). Subsequently, we determined the relationships among DNM3OS, miR-214-5p, and E2F2 in GC-1 spg cells. Results from luciferase reporter assays suggested that miR-214-5p mimics decreased the luciferase the activity of the WT-E2F2 reporter vector, while there was no significant change in the activity of the MUT-E2F2 reporter (Figure 4(c)). Next, qPCR (Figure 4(d)) and western blot analyses (Figure 4(e)) revealed that the levels of E2F2 expression in GC-1 spg cells were significantly reduced after ADR stimulation, and those decreases were notably reversed after DNM3OS knockdown.

### 3.5. Downregulation of miR-214-5p Inhibited the Apoptosis and Senescence of Spermatogonia Induced by ADR Treatment

**Figure 5:**
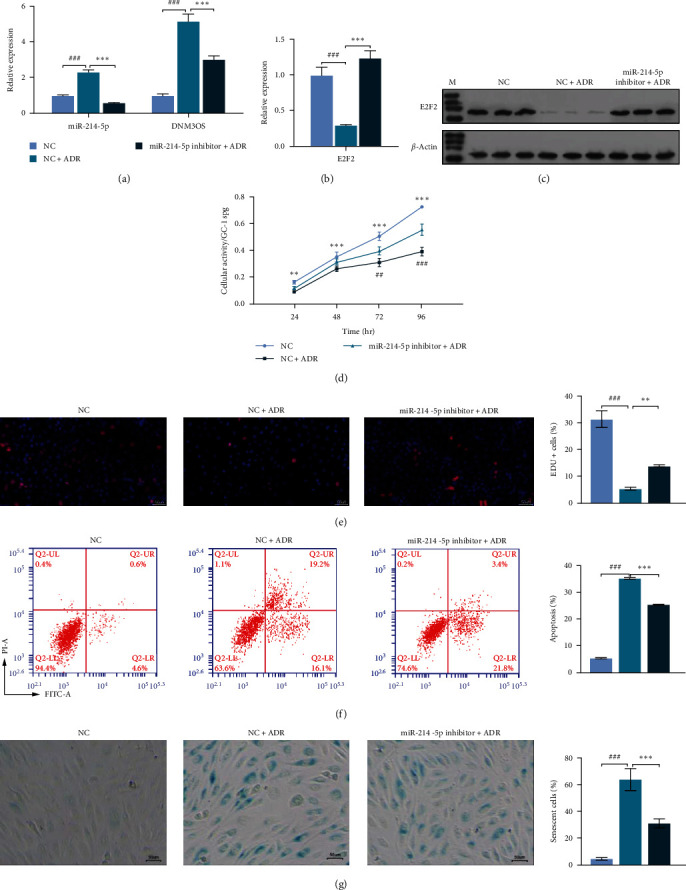
Downregulation of miR-214-5p decreased the apoptosis and senescence of spermatogonia induced by ADR treatment. GC-1 spg cells were treated with 0.5 *μ*M ADR for 24 hr and subsequently transfected with the miR-214-5p inhibitor or NC. (a) The levels of DNM3OS and miR-214-5p expression were determined by quantitative real-time PCR. (b) and (c) E2F2 expression at the mRNA and protein levels was determined. (d) Cellular activity was determined by the CCK-8 assay. (e) EDU incorporation assays were performed to assess cell proliferation ability. (f) Apoptotic cells were identified by flow cytometry with Annexin V-PI double staining. (g) Senescence-associated *β*-galactosidase (SA-*β*-gal) staining was used to evaluate cell senescence. Data represent the mean value ± SD. ^##^*p* < 0.01, ^###^*p* < 0.001 represents NC vs. NC + ADR;  ^*∗∗*^*p* < 0.01,  ^*∗∗∗*^*p* < 0.001 represents NC + ADR vs. miR-214-5p inhibitor + ADR.

We next performed loss-of-function assays to verify the regulatory effect of miR-214-5p in GC-1 spg cells. qPCR results suggested that the upregulation of miR-214-5p and DNM3OS expression in ADR-treated GC-1 spg cells was significantly reduced after miR-214-5p knockdown (Figure 5(a)). Under conditions of miR-214-5p knockdown, results also showed that E2F2 expression at both the mRNA (Figure 5(b)) and protein (Figure 5(c)) levels were significantly increased in ADR-treated GC-1 spg cells. Subsequent cell behavior testing revealed that the knockdown of miR-214-5p markedly reversed the impaired cellular activity (Figure 5(d)) and proliferation ability (Figure 5(e)) of GC-1 spg cells induced by ADR. The elevated levels of cell apoptosis (Figure 5(f)) and senescence (Figure 5(g)) induced by ADR were both obviously reduced after the downregulation of miR-214-5p.

## 4. Discussion

NOA is a complex type of male infertility caused by a spermatogenesis problem resulting from both testicular pathology and hormone abnormalities [32]. While many attempts have been made to understand the molecular pathogenesis of NOA, the epigenetic regulators of abnormal spermatogenesis remain unclear. LncRNAs comprise a class of endogenous ncRNAs that contain >200 nucleotides. Previous studies showed that lncRNAs participate in regulating human spermatogenic cell development [33]. Studies have also shown that miRNA-122-5P enhances the proliferation and DNA synthesis of human spermatogonial stem cells by targeting CBL, competing with lncRNA-CASC7, and inhibiting the early stage of apoptosis [22]. LncRNANLC1-C has a decreased inhibitory effect on sperm maturation in the testicular tissue of male infertility patients and is involved in the regulation of spermatogenesis as a competing endogenous RNA of miRNA-302A and miRNA-383 [34]. LncRNA AK015322 is highly expressed in spermatogonial stem cells and regulates the proliferation of spermatogonial stem cells through its role as a miRNA-19b-3p sponge [16]. However, our understanding of the functional role of lncRNA in NOA remains limited. In the current study, we preformed microarray analyses on testicular biopsies from patients with NOA and control subjects and identified 2,652 differentially expressed lncRNAs (1,656 upregulated and 996 downregulated genes). Data obtained by quantitative real-time PCR were fully consistent with the expression patterns of several top differentially expressed lncRNAs identified by microarray assays, thereby supporting the credibility and validity of the microarray results. Among the validated lncRNAs, DNM3OS was the most highly upregulated lncRNA in TCAM-2 and GC-1 spg cells undergoing treatment with either LPS or ADR alone. The dynamin 3 opposite strand (DNM3OS) is transcribed from the intron sequence of the dynamin 3 gene [35]. In normal development, malignant tumors, and various noncancerous diseases, DNM3OS functions at the junction of key pathways that regulate important molecular pathways and cellular processes [36]. DNM3OS expression is significantly increased in ovarian cancer tissue and cell lines, and the increase is associated with a poor prognosis, as it enhances the proliferation, migration, and invasion ability of ovarian cancer cells [37]. In a cellular model of Huntington's disease, the DNM3OS/miR-196b-5p/GAPDH pathway was found to be involved in the molecular pathogenesis of the disease [38]. DNM3OS expression is downregulated in patients with osteoarthritis, and its overexpression was found to inhibit the apoptosis of CHON-001 chondrocytes [39]. In addition, DNM3OS has been shown to promote the inflammatory response of macrophages in diabetes via an independent mechanism [40].

We also demonstrated that DNM3OS knockdown could alleviate the impaired cellular activity and proliferative ability, as well as the increases in apoptosis and senescence of mouse spermatogonial GC-1 spg cells induced by ADR treatment. Spermatogenesis is a highly complex process in which the proliferation and growth of spermatogonia allow for spermatocytogenesis, meiosis (I and II) production of haploid germ cells, and the generation of mature spermatozoa via numerous morphological changes of round spermatids [41, 42]. Defects in any of these complex processes can prevent the production of mature spermatozoa and induce the occurrence of NOA [43]. Based on the above findings, we speculated that in mice, DNM3OS mainly affected the spermatocytogenesis process in spermatogenesis by regulating spermatogonial GC-1 spg cell proliferation and growth. The ability of ADR treatment to induce apoptosis and senescence via a DNA damage response was attenuated after the knockdown of DNM3OS.

*DNM3OS*, a gene that is transcribed into a ncRNA, encodes for three miRNAs: miR-199a, miR-199a ^*∗*^, and miR-214 [44]. Watanabe et al. [31] found that *DNM3OS*, as a miRNA coding gene, is a precursor transcript of miR-214 [31]. Studies have shown that lncRNA DNM3OS can maintain chondrocyte proliferation independent of two cocistronic miRNAs: miR-199a and miR-214 [45]. As the product of *DNM3OS*, miR-214 directly inhibits CCN2 mRNA during activation of hepatic stellate cells [46]. Consistent with those results, our study showed that DNM3OS is a provider of pe-miR-214-5p, which positively regulates miR-214-5p expression in GC-1 spg cells. Our *in vitro* experiments indicated that the knockdown of miR-214-5p produced results similar to those of DNM3OS knockdown on ADR-induced apoptosis and senescence in GC-1 spg cells. Another study reported that transcription of miR-214-5p may originate from the intron sequence of DNM3OS [47]. Research studies have also suggested that miR-214-5p plays an important role in various cellular functions. Teng et al. [48] reported that lncRNA RNA component of mitochondrial RNA processing endoribonuclease (RMRP) enhanced hypoxia-induced injury in H9C2 cells by targeting miR-214-5p. Inhibition of MiR-214-5p was found to markedly attenuate antioxidant stress, inhibit apoptosis, and increase nerve fiber repair in a rat model of spinal cord injury [49]. In addition, miR-214-5p is involved in the protective effect of dexmedetomidine against neurological injury in Alzheimer's disease [50]. Subsequently, we screened the possible downstream genes of miR-214-5p and found that *E2F2* was one of those targets. Moreover, the knockdown of DNM3OS downregulated E2F2 expression in GC-1 spg cells undergoing ADR treatment. E2F2 has been shown to play an important role in regulating physiological processes such as the cell cycle, cell proliferation, DNA damage repair, and autophagy [46]. In renal cell cancer cells, E2F2 is the direct target of miR-214-5p, and lncRNA rcat1 can protect E2F2 from miR-214-5p-mediated degeneration [51]. The LncRNA DNR/miR-214-5P/E2F2 axis functions as an oncogene during pancreatic cancer development and is a potential target for pancreatic cancer therapy [52]. Circcul2 inhibits the proliferation, invasion, and migration of retinoblastoma cells by regulating the miR-214-5P /E2F2 axis [53]. In addition, among the activating E2F members, E2F2 is most highly concentrated in spermatocytes in the mid to late prophase of meiosis [54]. Our study found that DNM3OS could promote ADR-induced apoptosis and senescence of spermatogonia GC-1 spg cells by regulating the miR-214-5P /E2F2 axis, which is consistent with the results of previous studies.

Our study helps to elucidate the role of DNM3OS in the pathogenesis of NOA, but it also has certain limitations. First, an *in vitro* experimental cell model was used in this study, and the correlation between DNM3OS and NOA was not verified *in vivo*. Thus, there is a lack of *in vivo* experiments to support our research conclusions. Second, the lack of long-term follow-up clinical data in the study made it impossible to evaluate whether the expression level of DNM3OS is correlated with the prognosis of NOA patients, and its potential value for predicting the course and prognosis of NOA remains unclear. Finally, due to the small sample size of the study, it does not fully represent the diversity of the NOA patient population, and the universality of our conclusions needs to be verified in future studies.

In conclusion, our study used microarray assays to identify the expression profiles of lncRNAs and mRNAs involved in NOA pathogenesis. Further experiments suggested that DNM3OS might function as a positive regulator of ADR-induced spermatogonial GC-1 spg cell apoptosis and senescence by negatively regulating E2F2 expression via promoting the transcription of miR-214-5p. These findings reveal new molecular mechanisms and biological functions of DNM3OS in NOA cells and suggest new targets for gene therapy of male infertility.

## Figures and Tables

**Table 1:** Patients' clinical information was collected from testicular tissue (*x* ± *s*).

Individual	NOA (*n* = 9)	Normal tissues (*n* = 9)
Age	28.26 ± 5.42	29.74 ± 4.06
Somatic karyotype	46, XY	46, XY
Y Chromosome microdeletions	No	No
Secondary sexual	Normal	Normal
Testicular volume (mL)	8.24 ± 3.78	13.42 ± 5.54
Follicle-stimulating hormone (mIU/mL)	20.45 ± 5.24	5.34 ± 3.12
Luteinizing hormone	9.87 ± 5.12	4.34 ± 1.56
Prolactin	11.57 ± 4.81	8.95 ± 5.14
Estradiol II	23.54 ± 11.54	27.42 ± 8.16
Testosterone	3.54 ± 2.75	5.12 ± 1.87

**Table 2:** Primers for quantitative real-time PCR.

Gene	Forward (5′–3′)	Reverse (5′–3′)
DNM3OS	GTGCTCTGAAGTGTTGGACA	TGCAGTGCCTAGAGATGGTA
miR-199a-5p	ACACTCCAGCTGGGCCCAGTGTTCAGACTACC	CTCAACTGGTGTCGTGGA
miR-199a-3p	ACACTCCAGCTGGGACAGTAGTCTGCACATTG	CTCAACTGGTGTCGTGGA
miR-214-5p	ACACTCCAGCTGGGTGCCTGTCTACACTTGCT	CTCAACTGGTGTCGTGGA
miR-214-3p	ACACTCCAGCTGGGACAGCAGGCACAGACAGG	CTCAACTGGTGTCGTGGA
E2F2	ACCACCTACTACACTTCGCTT	GGAATTCAGGGACCGTAGG
U6	CTCGCTTCGGCAGCACA	AACGCTTCACGAATTTGCGT
GAPDH	TGTTCGTCATGGGTGTGAAC	ATGGCATGGACTGTGGTCAT

**Table 3:** Published papers on the downstream targets of miR-214-5p.

Target gene	Cell type	References
KLF12	Preadipocytes	Agarwal et al. [1]
TGF-*β*	Prostate cancer/bone marrow stem cells	Kuyucu et al. [2] and Watanabe et al. [31]
E2F2	Renal cell carcinoma/retinoblastoma/pancreatic cancer	Practice Committee of the American Society for Reproductive Medicine in collaboration with the Society for Male R, Urology [3], Cesana et al. [19], and Herman et al. [32]
CRMP5	Prostate cancer	Caroppo and Colpi [4]
KLF5	Hepatocellular carcinoma	Pena et al. [5]
ROCK1	Osteosarcoma/NSCLC	Ramasamy et al. [6] and Hu et al. [16]
DDX5	Osteosarcoma	Zhou et al. [7]
PPARGC1B	Osteosarcoma	Smith et al. [8]
SOX4	Osteosarcoma/prostate cancer/cervical cancer/colorectal carcinoma	Beermann et al. [9], Hu et al. [12], Hua et al. [21], and Dimova and Dyson [25]
C1QTNF1	PK15 cells	Joshi and Rajender [10]
ITGA7	Osteoclasts	Mukherjee et al. [11]
CXCR5	Microglia	Hu et al. [13]
FGF2	Keloid fibroblasts	Bo et al. [14]
BMP2	Bone marrow mesenchymal stem cells	Li et al. [15] and Faghihi et al. [18]
SEMA4C	Cervical cancer	Krol et al. [17]
TWIST1	JEG-3 cells	Arefnia et al. [20]
CIZ1	NSCLC	Zhou et al. [22]
smad4	Mesangial cells	Attwooll et al. [23]
JAG1	Colorectal cancer	Trimarchi and Lees [24]
FAS ligand	H9c2 cells	Wong et al. [26]
RAB14	Esophageal cancer	Helin [27]
CLIC4	Breast cancer	Iwasaki et al. [28]
CDC27	Glioma	Hong et al. [29]
TEAD1	Cardiomyocytes	Shu et al. [30]
COX20	Umbilical vein endothelial cells	Liang et al. [33]
zest 12	Hippocampal neurons	Lü et al. [34]
BCL11B	Peripheral blood mononuclear cells	Mitra et al. [35]

## Data Availability

All data are available from the corresponding author with reasonable request.
